# Calmodulin kinase II-dependent transactivation of PDGF receptors mediates astrocytic MMP-9 expression and cell motility induced by lipoteichoic acid

**DOI:** 10.1186/1742-2094-7-84

**Published:** 2010-11-24

**Authors:** Hui-Hsin Wang, Hsi-Lung Hsieh, Chuen-Mao Yang

**Affiliations:** 1 Department of Physiology and Pharmacology, Chang Gung University, Tao-Yuan, Taiwan; 2 Department of Nursing, Division of Basic Medical Sciences, Chang Gung Institute of Technology, Tao-Yuan, Taiwan

## Abstract

**Background:**

Lipoteichoic acid (LTA) is a component of Gram-positive bacterial cell walls, which has been found to be elevated in cerebrospinal fluid of patients suffering from meningitis. Moreover, matrix metalloproteinases (MMPs), MMP-9 especially, have been observed in patients with brain inflammatory diseases and may contribute to brain disease pathology. However, the molecular mechanisms underlying LTA-induced MMP-9 expression in brain astrocytes remain unclear.

**Objective:**

The goal of this study was to examine whether LTA-induced cell migration is mediated by calcium/calmodulin (CaM)/CaM kinase II (CaMKII)-dependent transactivation of the PDGFR pathway in rat brain astrocytes (RBA-1 cells).

**Methods:**

Expression and activity of MMP-9 induced by LTA was evaluated by zymographic, western blotting, and RT-PCR analyses. MMP-9 regulatory signaling pathways were investigated by treatment with pharmacological inhibitors or using dominant negative mutants or short hairpin RNA (shRNA) transfection, and chromatin immunoprecipitation (ChIP)-PCR and promoter activity reporter assays. Finally, we determined the cell functional changes by cell migration assay.

**Results:**

The data show that c-Jun/AP-1 mediates LTA-induced MMP-9 expression in RBA-1 cells. Next, we demonstrated that LTA induces MMP-9 expression via a calcium/CaM/CaMKII-dependent transactivation of PDGFR pathway. Transactivation of PDGFR led to activation of PI3K/Akt and JNK1/2 and then activated c-Jun/AP-1 signaling. Activated-c-Jun bound to the AP-1-binding site of the MMP-9 promoter, and thereby turned on transcription of MMP-9. Eventually, up-regulation of MMP-9 by LTA enhanced cell migration of astrocytes.

**Conclusions:**

These results demonstrate that in RBA-1 cells, activation of c-Jun/AP-1 by a CaMKII-dependent PI3K/Akt-JNK activation mediated through transactivation of PDGFR is essential for up-regulation of MMP-9 and cell migration induced by LTA. Understanding the regulatory mechanisms underlying LTA-induced MMP-9 expression and functional changes in astrocytes may provide a new therapeutic strategy for Gram-positive bacterial infections in brain disorders.

## Background

Bacterial infections are responsible for a number of inflammatory diseases including brain inflammation [1]. Gram-positive bacterial infections of the central nervous system (CNS) occur either as bacterial meningitis or as brain abscess, being localized to the membranes surrounding the brain or in its parenchyma, respectively [2]. Lipoteichoic acid (LTA), an amphiphilic polymer, is a component of the Gram-positive bacterial cell wall that induces glial inflammatory activation *in vitro *and *in vivo *[3,4]. For the initiation of LTA signaling, Toll-like receptors (TLRs), TLR2 especially are believed to be responsible for LTA recognition following challenge by Gram-positive bacteria such as *Staphylococcus aureus *and *Streptococcus pneumouniae *[5,6]. Upon binding to TLR heterodimers (*i.e. *TLR2/TLR1 or TLR2/TLR6 complex), LTA exerts a sequential activation of members of IL-1 receptor-associated kinase (IRAK) family and tumour-necrosis factor-receptor-associated factor 6 (TRAF6), mediated by a TLR adaptor protein MyD88. Ultimately, TLR signaling activates proteins of the NF-κB and MAPK families, leading to modulation of gene expression of cytokines and other pro-inflammatory proteins [7,8].

In the CNS, glial cells such as astrocytes and microglia are regarded as targets in Gram-positive bacterial infection [9,10]. Several lines of evidence suggest a causal relationship between LTA challenges and CNS diseases, which involves glial activation and TLR2 signaling [10-12]. In astrocytes of the CNS, TLR signaling has been shown to be involved in brain inflammatory changes [13,14], accompanied by up-regulation of several genes with pro-inflammatory and pro-apoptotic capabilities [11,15,16]. However, the role of astrocytes, the major regulator of fundamental biological functions of the CNS [17], in LTA-induced brain inflammation remains poorly defined.

Matrix metalloproteinases (MMPs), a zinc-dependent proteinases family, are involved in normal development and wound healing as well as in pathophysiological implications such as atherosclerosis and tumor metastasis. In brain, an increasing number of studies suggest an elevation of MMP-9 in various CNS diseases [18,19]. Moreover, pro-inflammatory factors, including cytokines, endotoxins, and oxidative stress, have been reported to up-regulate MMP-9 in astrocytes *in vitro *[20,21], indicating that during neuroinflammation, MMP-9 activity may be regulated by diverse factors in the CNS. Furthermore, a series of functional element-binding sites have been identified, including NF-κB, Ets, and AP-1 within the MMP-9 promoter [22], which can be induced by diverse stimuli. A recent report has shown that LTA increases MMP-9 expression via ERK pathway in RAW 264.7 macrophages [23]. Moreover, our studies have demonstrated that interleukin-1 (IL-1β), bradykinin (BK) and oxidized low-density lipoprotein (oxLDL) up-regulate MMP-9 expression via NF-κB, Elk-1, and AP-1 signalings in rat astrocytes [20,24,25]. However, the mechanisms underlying the regulation of MMP-9 expression by LTA in astrocytes are still unclear.

In response to pathogenic ligands, TLR2/MyD88 activates PI3K/Akt, MAPKs, and NF-κB pathways, which modulate immune responses following ligand recognition [26-28]. Moreover, activation of these signaling cascades and transcription factors has been reported to be involved in induction of MMP-9 in rat astrocytes [20,24,25]. Moreover, transactivation of receptor tyrosine kinases such as platelet-derived growth factor receptor (PDGFR) by several stimuli has also been implicated in mediating cellular functions of glial cells [29]. More recently, we have demonstrated that LTA induces MMP-9 expression via transactivation of PDGFR and activation of NF-κB in astrocytes [30]. Here, we further investigate the molecular mechanisms underlying LTA-induced MMP-9 expression in cultured RBA-1 cells. These findings demonstrate that in RBA-1 cells, LTA-induced MMP-9 expression is mediated through Ca^2+ ^signaling pathway, CaMKII-dependent transactivation of PDGFR, and PI3K/JNK/c-Jun (AP-1). Moreover, LTA-induced MMP-9 expression is positively associated with cell motility (migration) in the RBA-1 cell culture model.

## Methods

### Materials

DMEM/F-12 medium, FBS, and TRIzol were from Invitrogen (Carlsbad, CA, USA). Hybond C membrane and ECL western blotting detection system were from GE Healthcare Bio-sciences (Buckinghamshire, UK). MMP-9 antibody was from NeoMarker (Fremont, CA, USA). Phospho-CaMKII, phospho-JNK, and phospho-c-Jun antibody kits were from Cell Signaling (Danver, MA, USA). CaMKII, c-Jun, and phospho-PDGFR antibodies were from Santa Cruz (Santa Cruz, CA, USA). GAPDH antibody was from Biogenesis (Boumemouth, UK). BAPTA/AM, thapsigargin (TG), calmidazolium chloride (CaMI), KN-62, AG1296, LY294002, SP600125, and tanshinone IIA (TSIIA) were from Biomol (Plymouth Meeting, PA, USA). Bicinchoninic acid (BCA) protein assay reagent was from Pierce (Rockford, IL, USA). LTA (from *Staphylococcus aureus*), enzymes, and other chemicals were from Sigma (St. Louis, MO, USA).

### Cell culture

RBA-1 cells were used throughout this study. This cell line was originated from a primary astrocyte culture of neonatal rat cerebrum and naturally developed through successive cell passages [31]. Staining of RBA-1 with the astrocyte-specific marker, glial fibrillary acid protein (GFAP), showed over 95% positive staining. In this study, the RBA-1 cells were used within 40 passages that show normal cellular morphological characteristics and had steady growth and proliferation in the monolayer system. Cells were cultured and treated as previously described [32]. Primary astrocyte cultures were prepared from the cortex of 6-day-old Sprague-Dawley rat pups as previously described [24]. The purity of primary astrocyte cultures was assessed with the astrocyte-specific marker, GFAP, showing over 95% GFAP-positive astrocytes [30]. The cells were plated on 12-well plates and 10-cm culture dishes for MMP gelatin zymography and RT-PCR, respectively. The culture medium was changed every 3 days.

### MMP gelatin zymography

RBA-1 cells were made quiescent at confluence by incubation in serum-free DMEM/F-12 for 24 h. Growth-arrested cells were incubated with LTA at 37°C for the indicated times. When inhibitors were used, they were added 1 h prior to the application of LTA. Treatment of RBA-1 cells with pharmacological inhibitors or LTA alone had no significant effect on cell viability determined by an XTT assay (data not shown). The culture media were collected and centrifuged at 4°C to remove cells and debris, then each sample was mixed with equal amount of non-reduced sample buffer and electrophoresed on 10% SDS-PAGE containing 1 mg/ml gelatin as a protease substrate. Following electrophoresis, gels were placed in 2.7% Triton X-100 for 30 min to remove SDS, and then incubated with developing buffer (50 mM Tris base, 40 mM HCl, 200 mM NaCl, 5 mM CaCl_2_, and 0.2% Briji 35; Novex) at 37°C for 24 h on a rotary shaker. After incubation, gels were stained in 30% methanol, 10% acetic acid, and 0.5% w/v Coomassie brilliant blue for 10 min followed by destaining. Mixed human MMP-2 and MMP-9 standards (Chemicon) are used as positive controls. Gelatinolytic activity was manifested as horizontal white bands on a blue background. Because cleaved MMPs were not reliably detectable, only pro-form zymogens were quantified.

### Total RNA extraction and RT-PCR analysis

For RT-PCR analysis of MMP-9 mRNA expression, total RNA was extracted from RBA-1 cells as previously described [32]. The cDNA obtained from 0.5 μg total RNA was used as a template for PCR amplification. Oligonucleotide primers were designed based on Genbank entries for rat MMP-9 and β-actin. The following primers were used for amplification: for MMP-9, forward primer 5'-AGTTTGGTGTCGCGGAGCAC-3'; reverse primer 5'-TACATGAGCGCTTCCGGCAC-3'; for β-actin, forward primer 5'-GAACCCTAAGGCCAACCGTG-3'; reverse primer 5'-TGGCATAGAGGTCTTTACGG-3'. The amplification was performed in 30 cycles at 55°C, 30 s; 72°C, 1 min; 94°C, 30 s. PCR fragments were analyzed on 2% agarose 1× TAE gel containing ethidium bromide and their size was compared to a molecular weight markers. Amplification of β-actin, a relatively invariant internal reference RNA, was performed in parallel, and cDNA amounts were standardized to equivalent β-actin mRNA levels. These primer sets specifically recognize only the genes of interest as indicated by amplification of a single band of the expected size (754 bp for MMP-9 and 514 bp for β-actin) and direct sequence analysis of the PCR product.

### Preparation of cell extracts and western blot analysis

For experiments, cells were made quiescent at confluence by incubation in serum-free DMEM/F-12 for 24 h. Growth-arrested RBA-1 were incubated with LTA at 37°C for various times. When inhibitors were used, they were added 1 h before the application of LTA. The cells were rapidly washed with ice-cold phosphate-buffered saline (PBS), scraped, and collected by centrifugation at 1000 ' g for 10 min. The collected cells were lysed with ice-cold lysis buffer containing (mM): 25 Tris-HCl, pH 7.4, 25 NaCl, 25 NaF, 25 sodium pyrophosphate, 1 sodium vanadate, 2.5 EDTA, 2.5 EGTA, 0.05% (w/v) Triton X-100, 0.5% (w/v) SDS, 0.5% (w/v) deoxycholate, 0.5% (w/v) NP-40, 5 mg/ml leupeptin, 5 mg/ml aprotinin, and 1 PMSF. The lysates were centrifuged at 45,000 × g for 1 h at 4°C to yield the whole cell extract. The protein concentration was determined by the BCA reagents according to the instructions of the manufacturer. Samples from these supernatant fractions (30 mg protein) were denatured and subjected to SDS-PAGE using a 10% (w/v) running gel. Proteins were transferred to nitrocellulose (NC) membrane and the membrane was incubated successively at room temperature with 1% (w/v) BSA in Tween-Tris buffered saline (TTBS) for 1 h. The phosphorylation of CaMKII, PDGFR, JNK, and c-Jun was determined by Western blot using an anti-phospho-CaMKII, phospho-PDGFR, phospho-JNK, or phospho-c-Jun antibody used at a dilution of 1:1000 in TTBS. Membranes were washed with TTBS four times for 5 min each, incubated with a 1:2000 dilution of anti-rabbit horseradish peroxidase antibody for 1 h. The membrane was extensively washed with TTBS. The immunoreactive bands were detected by UVP Biospectrum^® ^imaging system (Upland, CA, USA).

### Measurement of intracellular Ca^2+ ^level

Intracellular Ca^2+ ^signaling was measured in confluent monolayers with the calcium-sensitive dye Fura-2/AM as described by Grynkiewicz *et al. *[33]. Upon confluence, the cells were cultured in serum-free DMEM/F-12 for 24 h before measurements were made. The monolayers were covered with 1 ml of DMEM/F-12 containing 5 μM Fura-2/AM and incubated at 37°C for 45 min. At the end of the period, the cover slips were washed twice with the physiological buffer solution containing (in mM): 125 NaCl, 5 KCl, 1.8 CaCl_2_, 2 MgCl_2_, 0.5 NaH_2_PO_4_, 5 NaHCO_3_, 10 HEPES, and 10 glucose, pH 7.4. The cells were incubated in physiological buffer for further 30 min to complete dye de-esterification. The cover slip was inserted into a quartz cuvette at an angle of approximately 45° to the excitation beam and placed in the temperature-controlled holder of a Hitachi F-4500 spectrofluorometer (Tokyo, Japan). Continuous stirring was achieved with a magnetic stirrer. Fluorescence of Ca^2+^-bound and unbound Fura-2 was measured by rapidly alternating the dual excitation wavelengths between 340 and 380 nm and electronically separating the resultant fluorescence signals at emission wavelength 510 nm. The autofluorescence of each monolayer was subtracted from the fluorescence data. The ratios (*R*) of the fluorescence at the two wavelengths are computed and used to calculate changes in intracellular Ca^2+ ^level.

### Plasmid construction, transient transfection, and promoter activity assay

The plasmids encoding dominant negative mutant of JNK (ΔJNK) and shRNA of CaMKII and c-Jun were provided by Dr. C.C. Chen (Department of Pharmacology, National Taiwan University, Taipei, Taiwan) and Dr. C.P. Tseng (Department of Medical Biotechnology and Laboratory Science, University of Chang Gung). The upstream region (-1280 to +19) of the rat MMP-9 promoter was cloned to the pGL3-basic vector containing the luciferase reporter system. Briefly, a 1.3-kb segment at the 5'-flanking region of the rat MMP-9 gene was amplified by PCR using specific primers from the rat MMP-9 gene (accession no. U36476): 5'-ccccggtaccGAAGGCGAAATGCTTTGCCC (forward/Kpn1) and 5'-ccccctcgaGGGTGAGAACCGAAGCTTCTG (reverse/Xho1). The pGL3-Basic vector, containing a polyadenylation signal upstream from the luciferase gene, was used to construct the expression vectors by subcloning PCR-amplified DNA of the MMP-9 promoter into the Kpn1/Xho1 site of this vector. The PCR products (pGL3-MMP-9WT) were confirmed by their size, as determined by electrophoresis, and by DNA sequencing. Additionally, the introduction of a mismatched primer mutation into the AP-1 to generate pGL3-MMP-9ΔAP-1 was performed, using the following (forward) primer: ΔAP-1: 5'-GCAGGAGAGGAAGCTGAGTTGAAGACA-3'. MMP-9-luc plasmid was transfected into RBA-1 cells. All plasmids were prepared by using QIAGEN plasmid DNA preparation kits. These constructs were transfected into RBA-1 cells, respectively, using a Lipofectamine reagent according to the instructions of manufacture. The transfection efficiency (~60%) was determined by transfection with enhanced GFP. After incubation with LTA (50 ng/ml), cells were collected and disrupted by sonication in lysis buffer (25 mM Tris, pH 7.8, 2 mM EDTA, 1% Triton X-100, and 10% glycerol). After centrifugation, aliquots of the supernatants were tested for luciferase activity using the luciferase assay system. Firefly luciferase activities were standardized for β-galactosidase activity.

### Chromatin immunoprecipitation assay

To detect the in vivo association of nuclear proteins with rat *mmp-9 *promoter, chromatin immunoprecipitation (ChIP) analysis was conducted as described previously [24]. RBA-1 cells in 100-mm dishes were grown to confluence and serum starved for 24 h. After treatment with LTA, protein-DNA complexes were fixed by 1% formaldehyde in PBS. The fixed cells were washed and lysed in SDS-lysis buffer (1% SDS, 5 mM EDTA, 1 mM PMSF, 50 mM Tris-HCl, pH 8.1) and sonicated on ice until the DNA size became 200~1000 base pairs. The samples were centrifuged, and the soluble chromatin was pre-cleared by incubation with sheared salmon sperm DNA-protein agarose A slurry (Upstate) for 30 min at 4°C with rotation. After centrifugation at 800 rpm for 1 min, one portion of the pre-cleared supernatant was used as DNA input control, and the remains were subdivided into aliquots and then incubated with a non-immune rabbit immunoglobulin G (IgG; Santa Cruz), anti-c-Jun (Santa Cruz), respectively, for overnight at 4°C. The immuno-precipitated complexes of Ab-protein-DNA were collected by using the above protein A beads, and washed successively with low-salt buffer (0.1% SDS, 1% Triton X-100, 2 mM EDTA, 20 mM Tris-HCl, pH 8.1, 150 mM NaCl), high-salt buffer (same as the low-salt buffer but with 500 mM NaCl), LiCl buffer (0.25 M LiCl, 1% NP-40, 1% deoxycholate, 1 mM EDTA, 10 mM Tris-HCl, pH 8.1), and Tris-EDTA (pH 8.0), and then eluted with elution buffer (1% SDS, 100 mM NaHCO_3_). The cross-linking of protein-DNA complexes was reversed by incubation with 5 M NaCl at 65°C for 4 h, and DNA was digested with 10 μg of proteinase K (Sigma)/ml for 1 h at 45°C. The DNA was then extracted with phenol-chloroform, and the purified DNA pellet was precipitated with isopropanol. After washing, the DNA pellet was resuspended in H_2_O and subjected to PCR amplification with the forward (5'-AGAGCCTGCTCCCAGAGGGC-3') and reverse (5'-GCCAAGTCAGGCAGGACCCC-3'), which were specifically designed from the distal AP-1 *mmp-9 *promoter region (-557 to -247). PCR products were analyzed on ethidium bromide-stained agarose gels.

### Migration assay

RBA-1 cells were cultured to confluence in 10-cm dishes and starved with serum-free DMEM/F-12 medium for 24 h. The monolayer cells were scratched manually with a blade to create extended and definite scratches in the center of the dishes with a bright and clear field. The detached cells were removed by washing the cells once with PBS. Serum-free DMEM/F-12 medium with or without LTA (50 μg/ml) was added to each dish as indicated after pretreatment with the inhibitors for 1 h, containing a DNA synthesis inhibitor hydroxyurea (10 μM) in the whole course. Images of migratory cells from the scratch boundary were observed and acquired at 0 and 48 h with a digital camera and a light microscope (Olympus, Japan). Numbers of migratory cells were counted from the resulting four phase images for each point and then averaged for each experimental condition. The data presented are summarized from three separate assays.

### Statistical analysis of data

Data were estimated using a GraphPad Prism Program (GraphPad, San Diego, CA, USA). Quantitative data were analyzed using ANOVA followed by Tukey's honestly significant difference tests between individual groups. Data were expressed as mean ± SEM. A value of *P *< 0.05 was considered significant.

## Results

### LTA-induced proMMP-9 expression is mediated through c-Jun/AP-1

**Figure 1:**
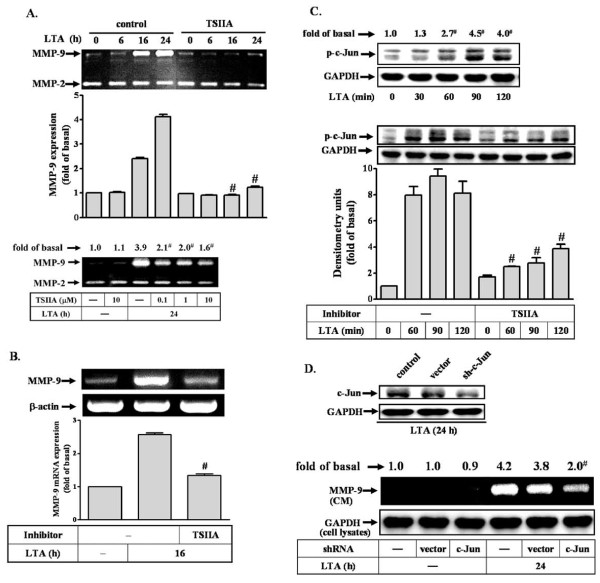
**c-Jun/AP-1 plays a critical role in LTA-induced MMP-9 expression**. (A) Time dependence of LTA-induced proMMP-9 expression and activity. Cells were pretreated with tanshinone IIA (TSIIA, 0.1, 1, or 10 μM) for 1 h and then incubated with 50 μg/ml LTA for the indicated time intervals. Conditioned media were collected and assayed for proMMP-9 expression and activity by gelatin zymography. ProMMP-2 expression is shown as an internal control. (B) Cells were pretreated with TSIIA for 1 h and then incubated with 50 mg/ml LTA for 16 h. Total RNA was extracted and analyzed by RT-PCR. (C) Time dependence of LTA-stimulated c-Jun/AP-1 phosphorylation. RBA-1 cells were pretreated with TSIIA for 1 h and then incubated with 50 mg/ml LTA for the indicated time intervals. Phosphorylation of c-Jun was determined by western blot using an anti-phospho-c-Jun (p-c-Jun) antibody. (D) Cells were transfected with a c-Jun shRNA plasmid for 48 h, and incubated with LTA for 24 h. The cell lysates were assayed by western blot using an anti-c-Jun antibody and anti-GAPDH antibody as a control (upper panel). Conditioned media (CM) and cell lysates were analyzed by zymographic analysis and western blot using an anti-GAPDH antibody as a control (lower panel). Data are expressed as mean ± SEM (A-C) or mean (A, C, D) of three independent experiments (n = 3). ^#^*P *< 0.01, as compared with the cells exposed to LTA alone. The figure represents one of three individual experiments.

Recently, we have demonstrated that LTA induces proMMP-9 up-regulation in astrocytes [30]. Moreover, MMP-9 promoter contains AP-1 binding sites that are essential for induction of several inflammatory genes such as MMP-9 [22,34]. Therefore, we first determined whether AP-1 was involved in LTA-induced proMMP-9 expression in RBA-1 cells, an AP-1 inhibitor tanshinone IIA (TSIIA) was used. The concentration of LTA at 50 μg/ml was used throughout this study according to our previous report (Hsieh et al., 2010) [30]. The conditioned media were collected and analyzed for *de novo *synthesis and activity of MMPs by gelatin zymography. As shown in Figure 1A, pretreatment with TSIIA (0.1-10 μM) significantly attenuated LTA-induced proMMP-9 expression and activity. Moreover, pretreatment with TSIIA (10 μM) also markedly inhibited LTA (50 μg/ml, 16 h)-induced MMP-9 mRNA expression, determined by RT-PCR (Figure 1B), suggesting that AP-1 is an important factor in LTA-induced proMMP-9 expression. To further determine whether an AP-1 subunit c-Jun was essential for LTA-induced proMMP-9 expression, cells were incubated with 50 μg/ml LTA for the indicated time intervals. As shown in Figure 1C (upper panel), LTA stimulated phosphorylation of c-Jun in a time-dependent manner. There was a significant increase within 60 min and reached a maximal response by 90 min. Pretreatment with TSIIA (10 μM) attenuated LTA-stimulated c-Jun phosphorylation (Figure 1C, lower panel). To confirm the crucial role of c-Jun in these responses, as shown in Figure 1D, transfection with c-Jun shRNA for 24 h down-regulated endogenous c-Jun protein expression (upper panel), and significantly attenuated LTA-induced proMMP-9 expression in RBA-1 cells (lower panel). These results suggested that LTA induces proMMP-9 expression via a c-Jun/AP-1 signal pathway.

### LTA-induced proMMP-9 expression requires JNK1/2 activation

**Figure 2:**
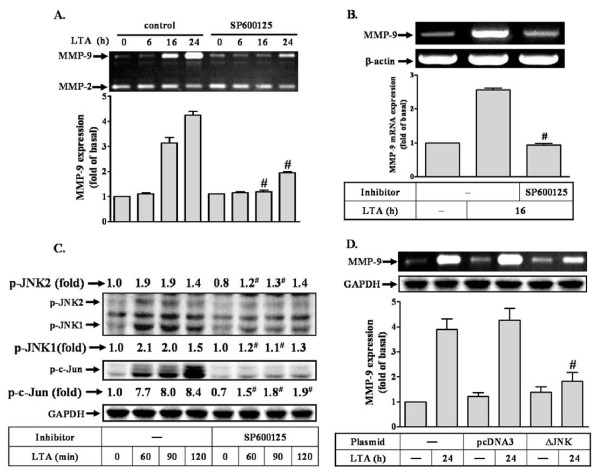
**LTA-induced c-Jun phosphorylation is mediated through JNK in RBA-1**. (A) Cells were pretreated with SP600125 (1 μM) for 1 h and then incubated with 50 μg/ml LTA for the indicated time intervals. Conditioned media were collected and assayed for proMMP-9 expression and activity by gelatin zymography. ProMMP-2 expression is shown as an internal control. (B) Cells were pretreated with SP600125 for 1 h and then incubated with 50 mg/ml LTA for 16 h. Total RNA was extracted and analyzed by RT-PCR. (C) Time dependence of LTA-stimulated JNK phosphorylation. RBA-1 cells were pretreated with SP600125 for 1 h and then treated with 50 mg/ml LTA for the indicated time intervals. Phosphorylation of JNK and c-Jun was determined by western blot using an anti-phospho-JNK or phospho-c-Jun antibody. (D) Cells were transfected with an empty vector (pcDNA3, as a control) or dominant negative mutant of JNK (ΔJNK) for 24 h, and then exposed to LTA (50 mg/ml) for 24 h. Cell lysates were assayed by western blot using an anti-GAPDH antibody as a control. Conditioned media were analyzed by zymographic analysis. Data are expressed as mean ± SEM (A, B, D) or mean (C) of three independent experiments (n = 3). ^#^*P *< 0.01, as compared with the cells exposed to LTA alone. The figure represents one of three individual experiments.

Several studies have demonstrated that JNK, a member of MAPK family, mediates up-regulation of MMP-9 in RBA-1 cells [20,25]. Thus, to investigate whether JNK1/2 also involved in LTA-induced proMMP-9 expression, a pharmacological inhibitor of JNK1/2, SP600125 was used. As shown in Figure 2A, pretreatment with SP600125 (1 μM) significantly inhibited the LTA-induced proMMP-9 expression during the period of observation. Moreover, LTA-induced MMP-9 mRNA expression was also significantly blocked by pretreatment with SP600125, determined by RT-PCR (Figure 2B). To further determine whether LTA-induced proMMP-9 expression was mediated through JNK1/2 phosphorylation, the kinetics of JNK1/2 phosphorylation stimulated by LTA was assessed by western blot using an anti-phospho-JNK1/2 antibody. As shown in Figure 2C, LTA stimulated JNK1/2 phosphorylation in a time-dependent manner with a maximal response within 60-90 min, which was significantly inhibited by pretreatment with SP600125 (1 mM) during the period of observation. Pretreatment with SP600125 (1 μM) also almost completely inhibited LTA-stimulated c-Jun phosphorylation (Figure 2C), suggesting that JNK was an upstream signal molecule of c-Jun/AP-1 cascade. Thus, to further ensure that JNK was involved in LTA-induced proMMP-9 expression, transfection of RBA-1 cells with a dominant negative mutant of JNK (ΔJNK) was performed. As shown in Figure 2D, transfection with ΔJNK markedly attenuated proMMP-9 induction by LTA. These results indicated that LTA-induced proMMP-9 expression is mediated through activation of JNK/c-Jun cascade in RBA-1 cells.

### Calcium-dependent signaling is involved in proMMP-9 induction by LTA

**Figure 3:**
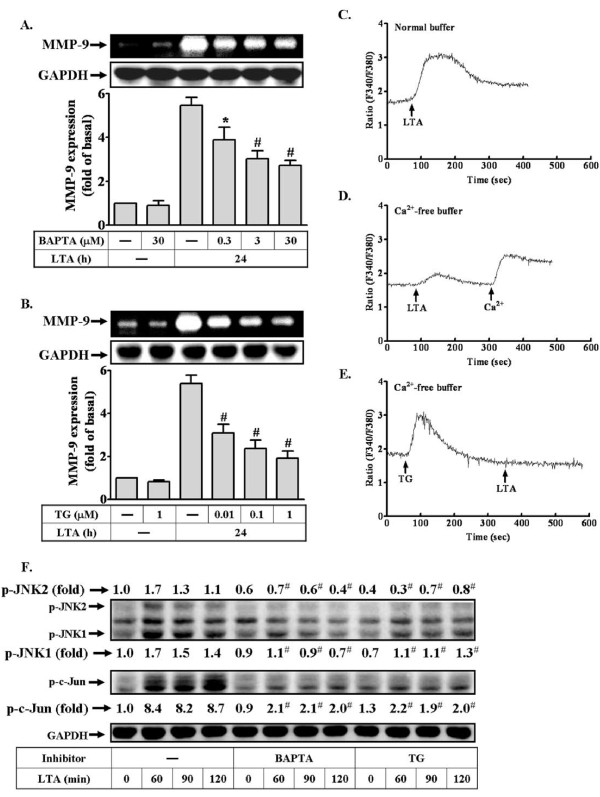
**LTA-induced Ca^2+ ^release from internal TG-sensitive Ca^2+ ^store plays a role in LTA-induced MMP-9 expression**. (A, B) Cells were pretreated with BAPTA/AM or TG for 1 h and then incubated with 50 μg/ml LTA for 24 h. Conditioned media were collected and analyzed by gelatin zymography. The cell lysates were assayed by western blot using an anti-GAPDH antibody as a control. (C-E) For Ca^2+ ^mobilization, confluent cultures of RBA-1 cells on glass coverslips were loaded with Fura-2/AM and fluorescent measurement of [Ca^2+^]_i _was carried out in a dual excitation wavelength spectrophotometer, with excitation at 340 nm and 380 nm. (C) Cells were incubated in Ca^2+^-containing normal buffer or (D) Ca^2+^-free buffer and then exposed to LTA at 100 s. (E) In a Ca^2+^-free buffer, cells were pretreated with 1 μM TG for 3 min, exposed to LTA at 100 s, and then 2 mM Ca^2+ ^was added to the cells. The figure represents one of at least five similar experiments. (F) Effects of calcium inhibitors on LTA-induced phosphorylation of JNK and c-Jun, RBA-1 cells were pretreated with BAPTA or TG for 1 h and then incubated with 50 mg/ml LTA for the indicated time intervals. Phosphorylation of JNK and c-Jun was determined by western blot using an anti-phospho-JNK or phospho-c-Jun antibody. Data are expressed as mean ± SEM (A, B) or mean (F) of three independent experiments (n = 3). *P < 0.05; ^#^*P *< 0.01, as compared with the cells exposed to LTA alone. The figure represents one of at least three individual experiments.

Furthermore, we examined which signaling molecules participated in activation of the JNK/c-Jun cascade and up-regulation of MMP-9 by LTA. A recent study has indicated that induction of MMP-9 by IL-1β is mediated through Ca^2+^-dependent signaling [35]. Hence, we investigated the role of intracellular Ca^2+ ^in LTA-induced proMMP-9 expression, the intracellular Ca^2+ ^chelator BAPTA/AM and ER Ca^2+^-ATPase blocker thapsigargin (TG) were used. As shown in Figures 3A and 3B, pretreatment with BAPTA/AM or TG for 24 h both significantly attenuated LTA-induced proMMP-9 expression in a concentration-dependent manner analyzed by zymography, suggesting that intracellular Ca^2+ ^signaling was required for LTA-induced proMMP-9 expression. Next, to determine whether LTA stimulated intracellular Ca^2+ ^signaling increase in RBA-1 cells, the intracellular Ca^2+ ^was measured by using a Ca^2+ ^indicator Fura-2/AM. The data showed that LTA rapidly stimulated an intracellular Ca^2+ ^increase in normal physiological buffer (Figure 3C). The sources of intracellular Ca^2+ ^increase may be ascribed to Ca^2+ ^release from intracellular stores and Ca^2+ ^influx from the extracellular fluid. Therefore, to differentiate these responses, the same experiments were performed in the Ca^2+^-free physiological buffer. As shown in Figure 3D, LTA also stimulated an intracellular Ca^2+ ^increase under Ca^2+^-free condition, but smaller than those of normal physiological buffer. Moreover, pretreatment with TG (1 μM) significantly blocked LTA-stimulated intracellular Ca^2+ ^increase under Ca^2+^-free physiological buffer (Figure 3E). These results indicated that the intracellular Ca^2+ ^increase by LTA may come from the intracellular TG-sensitive Ca^2+ ^stores and the extracellular Ca^2+ ^influx which is essential for LTA-induced proMMP-9 expression in RBA-1 cells.

To further determine the effect of Ca^2+ ^signaling on LTA-stimulated JNK/c-Jun cascade, the JNK and c-Jun phosphorylation stimulated by LTA in the presence of BAPTA/AM or TG were assessed by Western blot. As shown in Figure 3F, pretreatment with BAPTA/AM (30 μM) or TG (1 μM) both markedly attenuated LTA-stimulated phosphorylation of JNK and c-Jun, suggesting that intracellular Ca^2+ ^increase is crucial for phosphorylation of JNK/c-Jun stimulated by LTA in RBA-1 cells.

### LTA-induced proMMP-9 expression via calmodulin kinase II (CaMKII)-dependent manner

**Figure 4:**
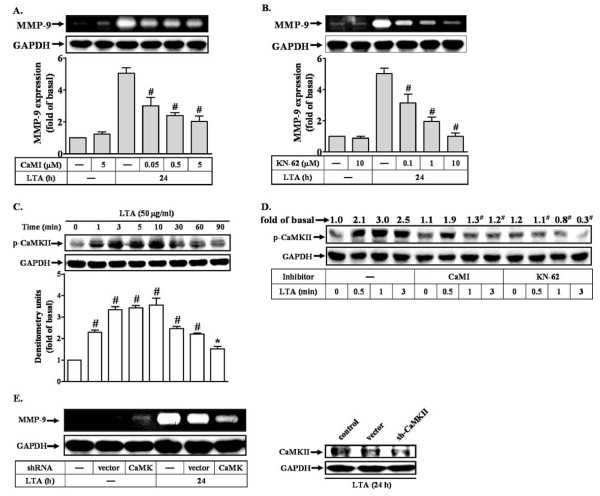
**Involvement CaMKII in LTA-induced MMP-9 expression in RBA-1**. (A, B) Cells were pretreated with inhibitors of calmodulin (CaMI) or CaM kinase II (KN-62) for 1 h and then incubated with 50 μg/ml LTA for 24 h. Conditioned media were collected and analyzed by gelatin zymography. Cell lysates were assayed by western blot using an anti-GAPDH antibody as a control. (C) Time dependence of LTA-stimulated phosphorylation of CaMK II. RBA-1 cells were incubated with 50 μg/ml LTA for the indicated time intervals. (D) Cells were pretreated CaMI or KN-62 for 1 h and then incubated with LTA for the indicated time intervals. Cell lysates were assayed by western blot using an anti-phospho-CaMKII antibody. The membranes were stripped and re-probed with anti-GAPDH antibody as a control. (E) Cells were transfected with a CaMKII shRNA plasmid for 48 h, and then incubated with LTA for 24 h. Cell lysates were assayed by western blot using an anti-CaMKII or anti-GAPDH (as a control) antibody. Conditioned media were collected and analyzed by gelatin zymography. Data are expressed as mean ± SEM (A-C) or mean (D) of three independent experiments (n = 3). *P < 0.05; ^#^*P *< 0.01, as compared with the cells exposed to LTA alone. The figure represents one of at least three individual experiments.

Several reports have indicated that CaMKII is a mediator between calcium signal and MAPK activation such as JNK [35,36]. To determine whether CaMKII was involved in LTA-induced proMMP-9 expression in RBA-1 cells, a CaMKII inhibitor KN-62 and its upstream molecule, calmodulin (CaM) inhibitor (CaMI) were used. These data showed that pretreatment with CaMI (Figure 4A) or KN-62 (Figure 4B) significantly inhibited LTA-induced proMMP-9 expression in a concentration-dependent manner, suggesting that CaM/CaMKII cascade is involved in LTA-induced proMMP-9 expression. We also determined whether LTA could stimulate CaMKII activation leading to MMP-9 expression. As shown in Figure 4C, LTA stimulated a time-dependent phosphorylation of CaMKII. A maximal response was obtained within 3 min and then declined within 60 min. LTA-stimulated CaMKII phosphorylation was significantly attenuated by pretreatment with CaMI (5 μM) or KN-62 (10 μM) during the period of observation, respectively (Figure 4D). To ascertain that CaMKII indeed participated in LTA-induced proMMP-9 expression, as shown in Figure 4E, transfection with CaMKII shRNA significantly knocked down the endogenous CaMKII protein expression (right panel) and attenuated LTA-induced proMMP-9 expression (Figure 4E, left panel), indicating that CaM/CaMKII was involved in LTA-induced proMMP-9 expression in RBA-1 cells.

**Figure 5:**
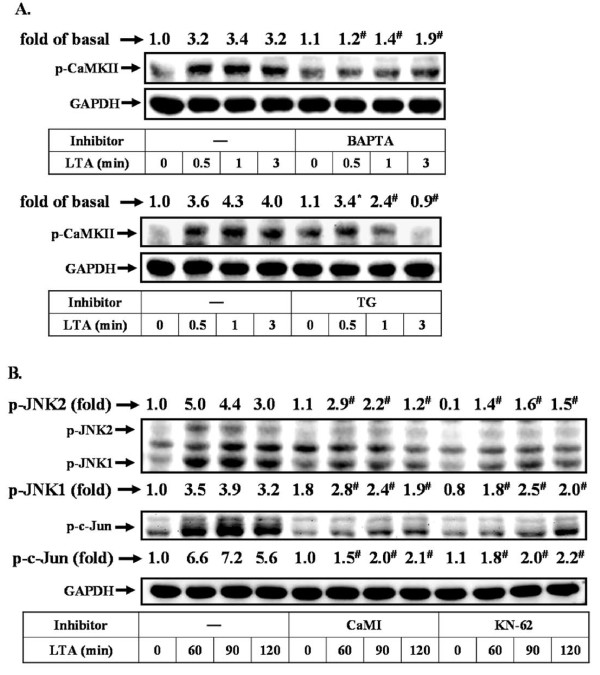
**Ca^2+^/CaMKII-dependent LTA-mediated activation of JNK and c-Jun in RBA-1 cells**. (A) Cells were pretreated with BAPTA (upper part) or TG (lower part) for 1 h and then incubated with LTA for the indicated time intervals. Cell lysates were assayed by western blot using anti-phospho-CaMKII antibody. (B) Cells were pretreated CaMI or KN-62 for 1 h and then incubated with LTA for the indicated time intervals. Cell lysates were assayed by western blot using an anti-phospho-JNK or phospho-c-Jun antibody. The membranes were stripped and re-probed with anti-GAPDH antibody as a control. Data are expressed as mean of three independent experiments (n = 3). *P < 0.05; ^#^*P *< 0.01, as compared with the cells exposed to LTA alone. The figure represents one of at least three individual experiments.

Next, to determine whether activation of CaMKII by LTA was mediated through Ca^2+ ^signaling, an upstream signaling molecule of CaM/CaMKII cascade, cells were pretreated with BAPTA/AM or TG for 1 h and then exposed to LTA (50 μg/ml) for the indicated time intervals. As shown in Figure 5A, LTA-stimulated CaMKII phosphorylation was significantly inhibited by pretreatment with BAPTA/AM (30 μM, upper panel) or TG (1 μM, lower panel) during the period of observation, suggesting that LTA stimulates phosphorylation of CaMKII via a Ca^2+^-dependent manner. Furthermore, to determine whether CaMKII mediated LTA-stimulated activation of JNK/c-Jun cascade, cells were pretreated with CaMI (5 μM) or KN-62 (10 μM) for 1 h and then treated with LTA (50 μg/ml) for the indicated time intervals. These data showed that LTA-stimulated phosphorylation of JNK and c-Jun was significantly inhibited by pretreatment of CaMI or KN-62 during the period of observation (Figure 5B). These results indicated that LTA-stimulated Ca^2+^-dependent CaM/CaMKII cascade was essential for JNK/c-Jun activation and MMP-9 expression in RBA-1 cells.

### LTA induces activation of JNK/c-Jun cascade via CaMKII-dependent transactivation of PDGFR

**Figure 6:**
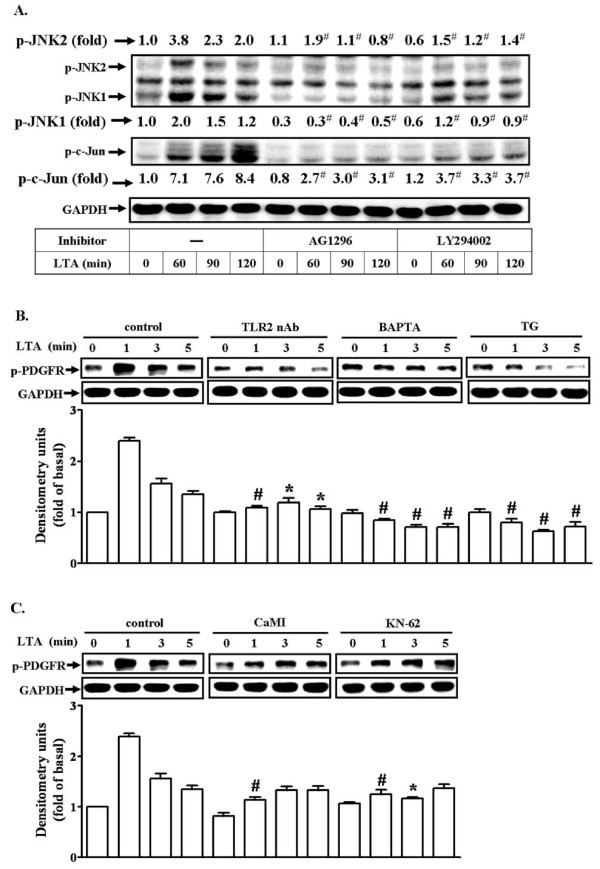
**LTA induces MMP-9 expression via Ca^2+^/CaMKII-dependent transactivation of PDGFR in RBA-1 cells**. (A) Cells were pretreated with AG1296 (10 μM) or LY294002 (30 μM) for 1 h and then incubated with 50 mg/ml LTA for the indicated time intervals. (B, C) Cells were pretreated with TLR2 neutralizing antibody (TLR2 nAb), BAPTA, TG, CaMI, or KN-62 for 1 h and then incubated with 50 mg/ml LTA for the indicated time intervals. Cell lysates were subjected to SDS-PAGE and blotted using an anti-phospho-JNK or anti-phospho-c-Jun (A) and anti-phospho-PDGFR (B, C) or anti-GAPDH (as an internal control) antibody. Data are expressed as mean ± SEM (B, C) or mean (A) of three independent experiments (n = 3). *P < 0.05; ^#^*P *< 0.01, as compared with the cells exposed to LTA alone. The figure represents one of at least three individual experiments.

Transactivation of growth factor receptor tyrosine kinases such as PDGFR has been shown to participate in glial cell functional changes induced by IL-1β [29]. Our recent study has also demonstrated that the PDGFR mediates LTA-induced proMMP-9 up-regulation in RBA-1 cells [30]. Therefore, we further examined whether activation of the PDGFR tyrosine kinase and its related signaling components were involved in LTA-stimulated JNK/c-Jun cascade. First, RBA-1 cells were pretreated with either AG1296 (a PDGFR tyrosine kinase inhibitor) or LY294002 (a PI3K inhibitor) for 1 h and then incubated with LTA for the indicated time intervals. We found that pretreatment with either 10 mM AG1296 or 30 mM LY294002 significantly inhibited LTA-induced JNK1/2 and c-Jun phosphorylation during the period of observation revealed by western blot (Figure 6A). To further ascertain that LTA-stimulated transactivation of PDGFR was mediated through a Ca^2+^-dependent CaMKII pathway, cells were pretreated with TLR2 neutralizing antibody (TLR2 nAb, 10 μg/ml), BAPTA/AM (30 μM), TG (1 μM), CaMI (5 μM), or KN-62 (10 μM), and then incubated with LTA for the indicated time intervals. As shown in Figures 6B and 6C, LTA stimulated a rapidly time-dependent phosphorylation of PDGFR, which was markedly attenuated by pretreatment with TLR2 nAb, BAPTA/AM, TG, CaMI, or KN-62, indicating that Ca^2+^/CaMKII-dependent transactivation of PDGFR cascade played a critical role in LTA-induced activation of JNK/c-Jun in RBA-1 cells.

### LTA induces c-Jun/AP-1 binding to the MMP-9 promoter and turned on MMP-9 transcriptional activity

**Figure 7:**
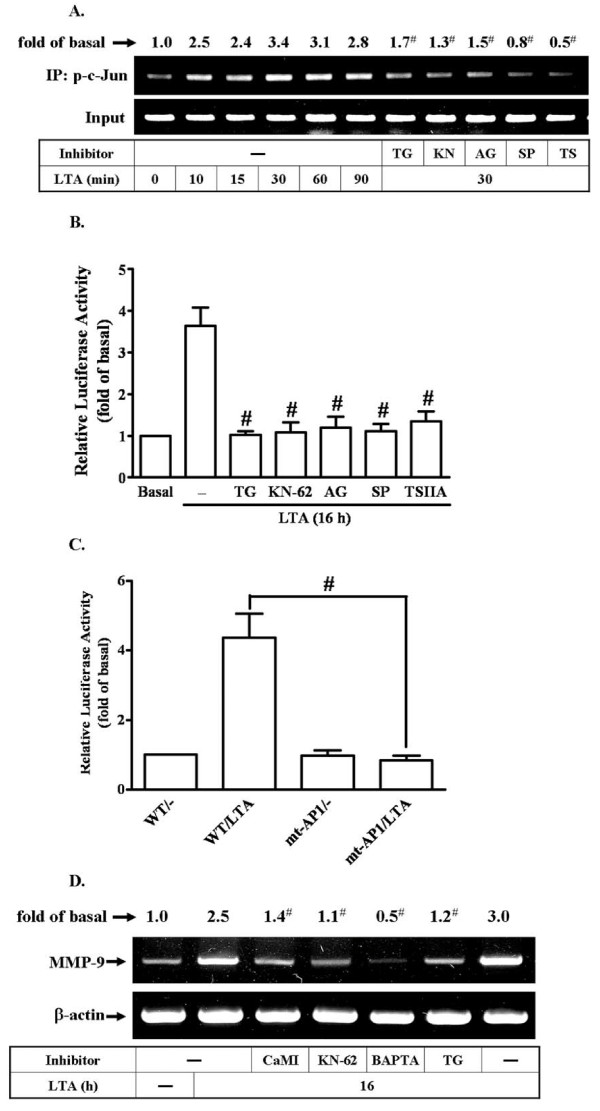
**c-Jun/AP-1 binding element is essential for LTA-induced MMP-9 expression through a Ca^2+^/CaMKII/PDGFR/PI3K/JNK pathway in RBA-1 cells**. (A) Time dependence of LTA-stimulated c-Jun/AP-1 binding activity. RBA-1 cells were incubated with 50 mg/ml LTA for the indicated time intervals, or cells were pretreated with TG, KN-62, AG1296, SP600125, or TSIIA for 1 h and then incubated with 50 mg/ml LTA for 30 min. c-Jun/AP-1 binding activity was analyzed by chromatin-IP (ChIP)-PCR assay. (B) Cells were transiently cotransfected with pGL-MMP9-Luc and pGal encoding for b-galactosidase for 24 h. The pGL-MMP-9-Luc-transfected cells were pretreated with TG, KN-62, AG1296, SP600125, or TSIIA for 1 h and then incubated with LTA for 16 h. (C) Activation of wild-type (WT) and AP-1-mutated (mt-AP1) MMP-9 promoter constructs by LTA. Cells were cotransfected with respective promoter constructs for 24 h and then incubated with 50 μg/ml LTA for 16 h. The values for beetle luciferase were normalized to that of b-galactosidase activity. (D) Cells were pretreated with CaMI, KN-62, BAPTA, or TG for 1 h and then incubated with LTA for 16 h. Total RNA were extracted and analyzed by RT-PCR. Data are expressed as mean ± SEM (B, C) or mean (A, D) of three independent experiments (n = 3). *P < 0.05; ^#^*P *< 0.01, as compared with the cells exposed to LTA alone. The figure represents one of at least three individual experiments.

Several studies have reported that the increase of MMP-9 gene expression is mediated through an AP-1-dependent pathway [37,38]. Moreover, rat MMP-9 promoter region contains AP-1 binding sites [22,34]. Hence, we used ChIP-PCR assay to determine whether c-Jun/AP-1 was involved in LTA-regulated MMP-9 gene expression. We first designed a pair of primers for MMP-9 promoter (-597 to -318), containing an AP-1 binding site. Chromatin was immunoprecipitated using an anti-c-Jun antibody, and the MMP-9 promoter region (-597 to -318) was amplified by PCR. As shown in Figure 7A, *in vivo *binding of phospho-c-Jun to the MMP-9 promoter was increased by LTA stimulation in a time-dependent manner. To further examine whether phospho-c-Jun binding is mediated through CaMKII-dependent PDGFR transactivation pathway, as shown in Figure 7A, LTA-induced phospho-c-Jun binding to the MMP-9 promoter was significantly inhibited by pretreatment with TG, KN-62, AG1296, SP600125, or TSIIA, analyzed by a ChIP-PCR assay, suggesting that Ca^2+^/CaMKII-dependent transactivation of PDGFR and JNK is involved in LTA-induced c-Jun/AP-1 binding to the MMP-9 promoter in RBA-1 cells.

Our recent report has demonstrated that LTA induces MMP-9 expression via regulating MMP-9 gene transcriptional activity [30]. Here, we further examined whether MMP-9 gene transcriptional activity was regulated by LTA via AP-1 in RBA-1 cells. The MMP-9 promoter was constructed into a pGL3-basic vector containing the luciferase reporter system (pGL-MMP-9-Luc), which contained AP-1 binding sites. To determine the effect of LTA on the MMP-9 promoter transcriptional activity, cells were cotransfected with these pGL-MMP-9-Luc construct and pGal and then pretreated with these inhibitors as mentioned above, following incubated with LTA (50 mg/ml) for 16 h. As shown in Figure 7B, pretreatment with TG (1 μM), KN-62 (10 mM), AG1296 (10 μM), SP600125 (1 μM), or TSIIA (10 μM) attenuated LTA-induced in MMP-9 promoter transcriptional activity, suggesting that LTA-induced in MMP-9 promoter transcriptional activity is mediated through Ca^2+^, CaMKII, PDGFR, JNK, and AP-1 in RBA-1 cells. To further ensure the AP-1 was critical for LTA-induced MMP-9 promoter activity via binding to AP-1 binding element on the MMP-9 promoter region, the wild-type (WT) MMP-9 promoter mutant by single-point mutation of the AP-1 binding site (mt-AP1) was constructed [29]. The data showed that LTA induced MMP-9 promoter transcriptional activity was significantly blocked in cells transfected with mt-AP1-MMP-9 reporter construct (Figure 7C), indicating that AP-1 binding element was required for LTA-induced MMP-9 promoter transcriptional activity. We further demonstrated that LTA-induced MMP-9 mRNA expression was also mediated through a Ca^2+^/CaMKII-dependent pathway determined by RT-PCR analysis (Figure 7D). These results confirmed that LTA-induced MMP-9 promoter activity is mediated through binding of activated c-Jun/AP-1 to the AP-1 element of the MMP-9 promoter region in RBA-1 cells.

### LTA enhances RBA-1 cell migration via up-regulation of proMMP-9

**Figure 8:**
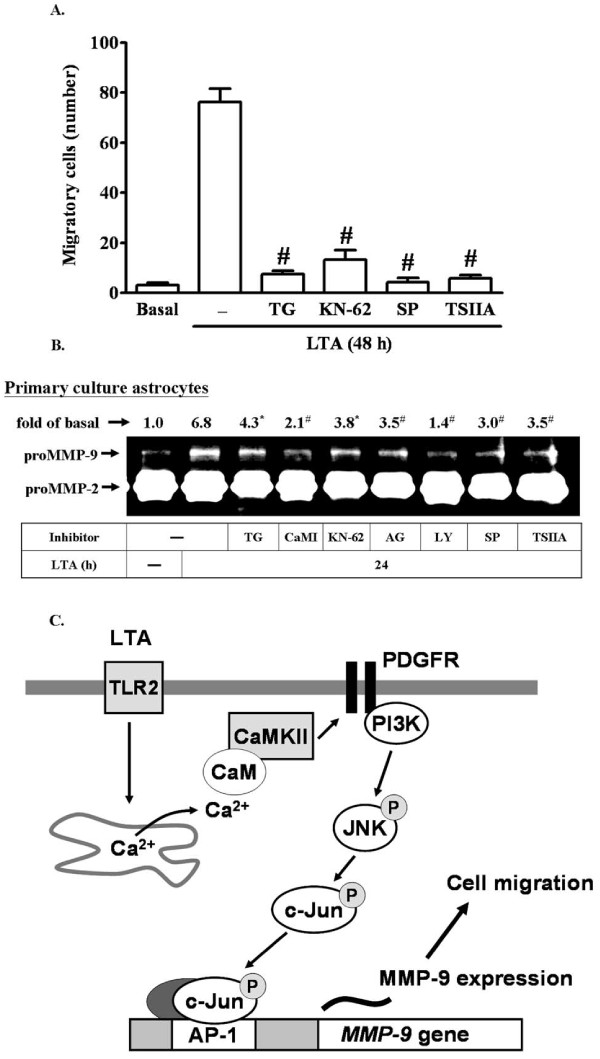
**LTA induces astrocytic migration through Ca^2+^/CaMKII-dependent c-Jun/AP-1 increasing MMP-9 expression**. RBA-1 cells were plated on coverslips, and grown to confluence. The coverslips were transferred to new 10-cm dishes containing serum-free medium for 24 h. The cells were pretreated with TG, KN-62, SP600125, or TSIIA (containing 10 μM hydroxyurea, a cell proliferation inhibitor) for 1 h and then incubated with 50 μg/ml LTA for 48 h. Phase contrast images of RBA-1 cells were taken at 48 h showing the response to LTA (n = 3). The number of LTA-induced cell migrations at 48 h were counted and summarized in (A). (B) LTA-induced MMP-9 expression via Ca^2+^/CaMKII-dependent PDGFR/PI3K/JNK/c-Jun cascade is observed in primary culture rat brain astrocytes. The rat primary culture astrocytes were pretreated with TG, CaMI, KN-62, AG1296, LY294002, SP600125, or TSIIA for 1 h and then incubated with LTA for 24 h. The conditioned media were analyzed by zymographic analysis. Data are expressed as mean ± SEM (A) or mean (B) of three independent experiments (n = 3). *P < 0.05; ^#^*P *< 0.01, as compared with the cells exposed to LTA alone. (C) Schematic representation of the LTA-mediated signaling pathways linked to proMMP-9 expression and cell migration in RBA-1 cells. Stimulation of LTA results in release of intracellular Ca^2+ ^from internal stores and activation of CaM/CaMKII-dependent transactivation of PDGFR and PI3K-JNK cascades, leading to activation of c-Jnu/AP-1. Activated c-Jun/AP-1 turns on MMP-9 gene expression and promotes cell motility of RBA-1 cells. This signaling pathway might contribute to sustained expression of MMP-9 which is required for RBA-1 cell migration.

Ultimately, to demonstrate the functional effect of proMMP-9 expression induced by LTA on RBA-1 cells, we evaluated cell migration of RBA-1 cells. The images of the RBA-1 cell migration were taken and counted at 48 h induction by LTA (50 μg/ml). The number of migratory RBA-1 cells was counted and the statistical data were presented in Figure 8A. We found that LTA-induced cell migration was significantly blocked by pretreatment with the inhibitors of intracellular TG-sensitive Ca^2+ ^stores (TG, 1 μM), CaMKII (KN-62, 10 mM), JNK (SP600125, 1 mM), or AP-1 (TSIIA, 10 mM), suggesting that up-regulation of proMMP-9 and its activity via CaMKII-dependent AP-1 pathway is required for enhancing cell migration induced by LTA in RBA-1 cells.

To further confirm whether this CaMKII-dependent AP-1 pathway mediated LTA-induced proMMP-9 expression also occurred in primary culture astrocytes, as shown in Figure 8B, pretreatment of primary cultured astrocytes with TG (1 μM), CaMI (5 μM), KN-62 (10 μM), AG1296 (10 μM), LY294002 (30 μM), SP600125 (1 μM), or TSIIA (10 μM) significantly attenuated 50 μg/ml LTA-induced proMMP-9 expression determined by gelatin zymography. These results demonstrated that in primary cultured astrocytes, up-regulation of proMMP-9 by LTA was, indeed, also mediated through a Ca^2+^/CaMKII-dependent PDGFR/PI3K/JNK/AP-1 pathway. These results of LTA-induced responses that appeared in RBA cells are similar to those of rat primary culture astrocytes.

## Discussion

The Gram-positive bacterium *Streptococcus pneumoniae *is the most common cause of acute bacterial meningitis worldwide [39,40], and reveals a close relationship between LTA challenge and CNS disease. The pathogenic progression involves glial activation and TLR2 signalings, which are linked to inflammatory neurodegeneration [9,10]. In the CNS, LTA exhibits detrimental effects on brain cellular functions, including induction of apoptosis, production of nitrosative and oxidative stresses, and disruption of BBB following group B *Streptococcus *or *Staphylococcus aureus *challenge [9,10,41]. The effects of MMP-9 on CNS diseases have been clarified by specific strategies of MMP-9 inhibition and clinical relevance [24,42,43]. Excessive MMP-9 activity is associated with sustained inflammation and BBB breakdown, leading to invasive brain injury. Blockade of MMP-9 activity by pharmacological inhibitors or gene knock-out strategies provides protective effects for the brain against cerebral ischemia [42,43]. However, the molecular mechanisms by which LTA induced MMP-9 expression in RBA-1 cells remain elusive. In this study, the mechanisms underlying LTA-induced proMMP-9 expression were established by using selective pharmacological inhibitors and transfection with dominant negative mutants coupling with gelatin zymography, western blotting and RT-PCR analyses. The requirement of transcription factors for the regulation of LTA-induced MMP-9 gene expression was determined by transfection of shRNA and reporter gene assay. These results suggest that in RBA-1 cells, activation of CaMKII-dependent PDGFR/PI3K/JNK linking to c-Jun/AP-1, mediated through a Ca^2+ ^signal, is essential for LTA-induced MMP-9 gene expression and cell migration.

Ca^2+ ^acts as a second messenger which impacts on a wide variety of physiological or pathological processes. Several external stimuli such as LTA stimulate respiratory burst in peripheral blood monocytes via increase intracellular Ca^2+ ^concentration [44]. In astrocytes, IL-1b can induce an immediate rise in intracellular free Ca^2+ ^concentration under normal conditions [45,46], which may contribute to up-regulating proMMP-9 expression [35]. Thus, we observed Ca^2+ ^responses to LTA and its effect on MMP-9 expression in RBA-1 cells. First, our data showed that LTA-induced proMMP-9 expression was attenuated by pretreatment with Ca^2+ ^chelator BAPTA/AM and ER Ca^2+^-ATPase inhibitor TG (Figures 3A and 3B), suggesting that intracellular Ca^2+ ^signal plays an important role in LTA-induced proMMP-9 expression. Next, we found that LTA stimulated a transient and rapidly intracellular Ca^2+ ^elevation (Figure 3C). The Ca^2+ ^responses reflect Ca^2+ ^mobilization from intracellular Ca^2+ ^stores and/or extracellular Ca^2+ ^influx from the extracellular fluid. We further demonstrated that LTA stimulates a transient increase of [Ca^2+^]_i _from the TG-sensitive intracellular Ca^2+ ^stores such as ER by using a Ca^2+^-free physiological buffer and pretreatment with TG (ER Ca^2+^-ATPase inhibitor) (Figures 4D and 4E), consistent with the report showing that LTA can stimulate intracellular Ca^2+ ^rise in tracheal smooth muscle cells [47]. Our data also showed that LTA-stimulated JNK-c-Jun/AP-1 pathway is mediated through Ca^2+ ^responses (Figure 3F), consistent with that activation of JNK/c-Jun by IL-1β is mediated through increased intracellular Ca^2+ ^in astrocytes [35]. These results suggest that Ca^2+ ^release from the TG-sensitive intracellular Ca^2+ ^stores may play a critical role in regulation of LTA-induced proMMP-9 expression in RBA-1 cells.

Calmodulin (CaM) is a key downstream component responding to Ca^2+ ^signal. Following binding to Ca^2+^, CaM undergoes a conformational change that renders it active and able to induce phosphorylation of CaM kinase II (CaMKII). Since to LTA can stimulate intracellular Ca^2+ ^increase, we hypothesized that LTA could activate the CaM/CaMKII pathway that results in proMMP-9 expression in RBA-1 cells. A previous report showed that several stimuli can activate a Ca^2+^-dependent phosphorylation of CaMKII [48], which may mediate MMPs expression in various cell types. For example, in osteoblastic cells, parathyroid hormone induces rat collagenase mRNA up-regulation through CaMKII activation [49]. Our data show that CaM and CaMKII participated in LTA-induced proMMP-9 expression by pretreatment with their respective inhibitors (Figures 4A and 4B). Moreover, we demonstrated that LTA can truly stimulate CaMKII phosphorylation (Figure 4C) which is mediated through Ca^2+ ^(Figure 5A) and CaM (Figure 4D). LTA-induced CaMKII-dependent MMP-9 expression was confirmed by transfection of cells with CaMKII shRNA (Figure 4E). Furthermore, we demonstrated that CaM/CaMKII cascade is involved in LTA-stimulated JNK-c-Jun/AP-1 activation (Figure 5B), consistent with the idea that MMP-9 induction by IL-1β is mediated through the CaM/CaMKII system in astrocytes [35]. For astrocytes, we show for the first time that LTA stimulates intracellular Ca^2+ ^increases from TG-sensitive Ca^2+ ^stores (*e.g. *ER), which participate in LTA-induced CaMKII phosphorylation and MMP-9 expression.

Several *in vivo *studies have indicated that PDGFR may play an important role in brain pathophysiology. Recent studies have suggested that transactivation of PDGFR contributes to MMP-9 up-regulation by IL-1β [29] or LTA [30] in RBA-1 cells. Several kinases, such as c-Src or PKCs, have been implicated in transactivation of EGFR or PDGFR [29,50], which in turn activate downstream PI3K/Akt and ERK1/2 cascades in various cell types including glial cells [29]. Several lines of evidence have also shown that MAPKs and PI3K/Akt mediate up-regulation of MMP-9 by various stimuli in brain astrocytes [25,29,32]. Our more recent data have demonstrated that IL-1β or LTA induces MMP-9 expression via sequential activation of c-Src-dependent transactivation of PDGFR linking to PI3K/Akt and ERK1/2 cascade in RBA-1 cells [29,30]. Here, we further demonstrate a novel pathway of PDGFR transactivation that may be shared by LTA/TLR system shown to interact with Ca^2+^-dependent CaM/CaMKII signaling, which also contributed to MMP-9 upregulation in astrocytes. It is consistent with the findings that μ-opioid receptor-mediated ERK activation involves CaM-dependent EGFR transactivation [51].

Accumulating evidence has shown that MMP-9 is upregulated via an AP-1-dependent manner in various cell types [25,35,52]. AP-1 is an important mediator to regulate MMP-9 gene expression upon different stimulation [38,53]. Upon stimulation, c-Jun could dimerize with c-Jun or c-Fos to form stable homodimers or heterodimer AP-1 that binds to a specific AP-1 site in the promoter region of target genes and enhances gene transcription [54]. Therefore, we investigated the role of c-Jun, an AP-1 subunit, in LTA-induced proMMP-9 expression. Our data show that c-Jun phosphorylation is essential for LTA-induced proMMP-9 expression (Figure 1). We found that pretreatment with AP-1 inhibitor (TSIIA) attenuated LTA-induced c-Jun phosphorylation and MMP-9 mRNA and protein levels. Such inhibitory effects were also achieved by transfection with shRNA for c-Jun on proMMP-9 expression (Figure 1D). Theses results are consistent with the known mechanisms of MMP-9 expression in human breast cancer cells [52] and astrocytes [25,35].

Moreover, AP-1 binding sites have been identified in the MMP-9 gene promoter [55], which might explain the modulation of exerted by LTA through AP-1 activation. To examine whether c-Jun/AP-1 indeed binds to AP-1 sites in the promoter region of MMP-9 gene, the binding activity of c-Jun/AP-1 was determined by a ChIP-PCR assay. The data show that LTA enhances phospho-c-Jun binding to MMP-9 promoter in a time-dependent manner (Figure 7A), consistent with the responses of phosphorylation of c-Jun (Figure 1C). Pretreatment of RBA-1 cells with TG, KN-62, AG1296, SP600125, or TSIIA markedly inhibited LTA-stimulated c-Jun/AP-1 binding to MMP-9 promoter (Figure 7A). These results suggest that LTA-stimulated c-Jun/AP-1 binding activity is mediated via a Ca^2+^/CaMKII-dependent JNK/c-Jun cascade, consistent with the idea that CaMKII-dependent JNK/c-Jun activation is involved in cytokine-stimulated AP-1 activation [35,56]. Subsequently, we also demonstrated LTA-stimulated MMP-9 transcriptional activity via the same pathway using a wild-type rat MMP-9 promoter-luciferase reporter plasmid (pGL-MMP-9-Luc) construct (Figure 7B). We further confirmed that the AP-1 binding site within the MMP-9 promoter is required for LTA-induced MMP-9 transcriptional activity by mutation of the AP-1 element on MMP-9 promoter activity (Figure 7C). Furthermore, our RT-PCR data also demonstrate that Ca^2+^-dependent CaMKII signaling molecules are involved in LTA-induced MMP-9 gene upregulation in RBA-1 cells (Figure 7D). These results indicate that LTA-induced MMP-9 expression is mediated through Ca^2+^/CaMKII-dependent PDGFR/PI3K/JNK pathway, associated with activation of transcription factor c-Jun/AP-1 in RBA-1 cells. Consistently, we also confirmed that rat brain primary culture astrocytes use the same identified signaling molecules to express MMP-9 under LTA stimulation (Figure 8B).

Cell motility is a fundamental process during embryonic development, wound healing, inflammatory responses, and tumor metastasis [57]. It has been reported that MAPKs, NF-κB, AP-1, and MMP-9 [24,25,58] contribute to cell motility in different cell types. Recently, we have demonstrated that a c-Src-dependent NF-κB pathway mediates LTA-induced MMP-9 expression and cell migration in RBA-1 cells [30]. Here, we further demonstrate that a Ca^2+^/CaMKII-dependent JNK/c-Jun pathway is involved in LTA-induced cell migration in RBA-1 cells (Figure 8A). Therefore, we suggest that upregulation of MMP-9 by LTA is essential for enhancing RBA-1 cell migration which is mediated through the Ca^2+^/CaMKII-dependent JNK/c-Jun pathway.

## Conclusion

These results demonstrate that LTA induces MMP-9 expression via sequential activation of Ca^2+^, CaMKII, PDGFR, PI3K, JNK, and transcription factor c-Jun/AP-1, leading to the promotion of astrocytic migration. Based on observations from the literature and on our findings, we depict in Figure 8C a model for Ca^2+^/CaMKII-dependent transactivation of the PDGFR pathway implicated in LTA-induced MMP-9 expression in RBA-1 cells. Although the molecular basis of CaMKII function in synaptic and behavioral memory is well established, the role of CaMKII during acute or chronic brain inflammation is still unclear. We demonstrate that CaMKII is a pivotal kinase linking Ca^2+ ^signal and JNK, and that this leads to expression of MMP-9 in CNS infection. Pharmacological approaches targeting MMP-9 and their specific upstream signaling components should yield useful therapeutic targets for CNS inflammatory diseases upon infection with Gram-positive bacteria.

## Competing interests

The authors declare that they have no competing interests.

## Authors' contributions

HHW designed and performed experiments, acquisition and analysis of data, and drafted the manuscript. HLH and CMY have co-conceived of the study, participated in its design and coordination, have been involved in drafting the manuscript and revising it critically for important intellectual content and have given final approval of the version to be published. All authors have read and approved the final version of this manuscript.
